# Explaining the unexplained in clinical infectious diseases: looking forward.

**DOI:** 10.3201/eid0403.980312

**Published:** 1998

**Authors:** B A Perkins, D Relman

**Affiliations:** Centers for Disease Control and Prevention, Atlanta, Georgia, USA.


### Explaining the Unexplained in Clinical Infectious Diseases: Looking Forward

**Bradley A. Perkins* and David Relman†**
*Centers for Disease Control and Prevention, Atlanta, Georgia, USA; and
†Palo Alto VA Medical Center, Palo Alto, California, USA

We examined the need to improve our ability to explain the unexplained in clinical infectious diseases, primarily through improvements in diagnostic technology. Part of the motivation for this effort came from an Emerging Infectious Disease Program (funded by the National Center for Infectious Diseases, Centers for Disease Control and Prevention [CDC]) to conduct surveillance for unexplained deaths and critical illnesses due to possibly infectious causes. This project has found that the number of such patients in the United States is substantial and that a probable causative agent can be identified in only a small fraction of these patients.

John Bartlett, Johns Hopkins University, Baltimore, Maryland, and Sherif Zaki, CDC, Atlanta, Georgia, addressed the current status and offered their perspectives on pneumonia, particularly acute respiratory distress syndrome (ARDS) and hemorrhagic pneumonia, syndromes frequently associated with unexplained critical illness. Greg Kovacs, Stanford University, Stanford, California, and Michael Eisen, Stanford University School of Medicine, Stanford, California, presented possible technologies and approaches to improving diagnostic capabilities—a sensitive biologic detection system (for toxins and host gene expression responses) for diagnosing infectious diseases.

### Pneumonia—Evolving Diagnostic Practices

Pneumonia, the most common infectious cause of death in the United States, accounts for approximately 45,000 deaths annually. In large hospital-based studies, no causative agent can be identified in 35% of community-acquired pneumonia cases. In actual practice, this proportion is probably 50% to 75%.

Over the last three decades, the proportion of community-acquired pneumonia cases in which *Streptococcus pneumoniae* was isolated has substantially declined. In the 1970s, the proportion was 62%; in the 1980s, 40%; and in 1991, 18%. Why has the recovery of pneumococci from patients with community-acquired pneumonia changed so dramatically? Have the causes of community-acquired pneumonia changed? Standard "gumshoe" microbiology to isolate pneumococci has taken a devastating hit in the 1990s due to outsourcing of microbiology services or just decreased emphasis on standard microbiology practices (e.g., collection and handling of clinical specimens). Newly recognized agents such as *Legionella pneumophila* may also explain some of the decrease in the proportion of pneumococci isolated.

Recommendations for the evaluation and management of community-acquired pneumonia, developed by the Infectious Disease Society of America, were published in the April issue of Clinical Infectious Diseases. These recommendations detail diagnostic tests as well as inadequacies in diagnostic technologies for several of the common causes of community-acquired pneumonia, including *Chlamydia pneumoniae*, *L. pneumophila*, and *Mycoplasma pneumoniae*.

### Pathologic Approach to the Diagnosis of Infectious Causes of Pulmonary Hemorrhage and Acute Respiratory Distress Syndrome

Pathologists should recognize patterns of tissue injury (especially in the lung parenchyma) that react in specific and predictable ways. This approach narrows diagnostic options and focuses testing efforts. Acute lung injury (e.g., diffuse alveolar damage or ARDS) and air space filling patterns (e.g., hemorrhage and pulmonary edema) of lung injury are two important patterns manifesting infectious disease. Examples include diffuse alveolar damage associated with adenovirus infection (smudge cells may be seen); measles (giant cells); respiratory syncytial virus (RSV) infection; influenza infections; Rocky Mountain spotted fever; typhus; legionnella; mycoplasma; and hemorrhage associated with aspergillosis, mucormycosis, leptospirosis, dengue, yellow fever, Lassa, and Ebola virus infection. The recognition of these patterns (combined with application of special stains, immunohistochemical reagents, and in situ hybridization) is a powerful tool in the diagnosis of unexplained critical infectious diseases.

Two examples of the application of these combined methods to the identification of infectious agents are the 1993 hantavirus epidemic in the southwestern United States and the 1995 leptospirosis epidemic in Nicaragua. In the hantavirus epidemic, healthy young adults contracted fever and rapidly progressive pulmonary disease consistent with ARDS, and many died within days of the onset of illness. Testing for a wide variety of agents was negative. Lung tissue showed interstitial pneumonitis and interalveolar edema; these patterns were consistent with viral pneumonia or toxic change. After serum samples from these patients were found to cross-react with known hantaviruses, antibodies were used to demonstrate hantavirus in the lung, kidney, and muscle tissues. In the leptospirosis epidemic, after heavy rains in northern Nicaragua, a number of persons became ill with fever, headache, muscle aches, hemorrhage, and severe ARDS; no prominent renal or hepatic manifestations were observed. Initial testing focused on hantavirus, dengue, and other viral agents, but results were negative. Pathologic examination of tissue from fatal cases showed pulmonary hemorrhage and diffuse alveolar damage, as well as renal and hepatic changes. In the 1980s, reports of leptospirosis epidemics in Korea and China prompted investigators to develop an immunohistochemical test for leptospirosis; the disease was subsequently found in kidney, liver, and lung specimens of Nicaraguan patients.

### Novel Bioassay for Detecting Toxin-Mediated Illness

The impetus for this project has been twofold: military detection of chemical and biological warfare agents and pharmaceutical screening. Cells are cultured on silicon chips, and their response to toxins is monitored in several ways (e.g., action potential for electrically active cells, impedance, and motility). These systems complement other approaches because they allow detection of unknown or unrecognized toxins. A cell monolayer is incredibly responsive because of its diffusion characteristics; this responsiveness can be tuned by selection of cells and through engineering. The use of cocultures can allow diversity in detection and response characteristics. In addition to detecting chemical and biological warfare agents, these systems can screen for antidotes by challenging the system with the toxin and adding a putative antidote. Pharmaceutical companies are interested in using this system for early screening of drug actions on cell physiology.

Chick myocardial cells and NG108 neuroblastoma hybrid cell lines were used to examine the shape and frequency of action potentials. Exposure of these cells to agents with known effects on cell physiology (e.g., epinephrine, verapamil, and tetrodotoxin) causes predictable changes (depending on the interaction of these toxins with transmembrane channels) on the shape of action potential curves when deviation from baseline is used as the internal control on response. Impedance measurement (alteration in electrical current after passage through a cell) can also be used to reflect changes in the cell membrane as a result of exposure to a toxin. The effect of toxins on the cytoskeleton can also be measured by cell motility through impedance. When this technology was first developed, it required approximately 1 $m^3$ of electronics support, but with silicon chips a laptop computer can now support the operation. A Windows application can handle the data processing, and the technology can be transferred to other laboratories.

### Cellular Scouts: Genome-Wide Expression Monitoring of Peripheral Blood to Detect and Characterize Pathogens

Using an easily constructed robot, we have been building DNA microarrays in which each dot represents different open reading frames. In the fully sequenced genome of *Saccharomyces cerevisiae,* there are 6,200 dots or open reading frames. The Human Genome Project has identified approximately 50,000 distinct cDNA sequences, and we have been using microarrays with approximately 15,000 of these. By the end of 1998, we will have all 50,000 genes on a microarray. For these assays we use a control and an experimental sample. From these samples, we isolate polyadentylated RNA by using any of a variety of kits, and then make fluorescently labeled cDNA copies, with each of the two samples labeled with a different color (e.g., one green and the other red). RNA is degraded to avoid any confounding signals; the samples are mixed and then hybridized to the microarray. The microarray is imaged by using a scanning laser confocal microscope, and through a process of quantitation, the relative representation of every gene in sample 1 versus sample 2 is calculated. These data provide a very high resolution fingerprint of what is going on in any cell(s) of interest. So when a sample from a healthy person is compared with one from an ill person, differences in gene expression should be sufficiently unique to diagnose particular infections.

First, however, we would like to know that cells respond to internal and external stimuli by at least some differences in expression of their genes, that specific stimuli result in distinct gene expression patterns, and that the response to stimuli evolves in a stereotypic temporal manner. Ideally, we would like not only to diagnose a particular infection but also to determine the stage of that infection. Preliminary data from our laboratory support these hypotheses. The patterns of human gene response to different stimuli, including T-cells stimulated with mitogens, cells exposed to DNA damaging agents, and cells infected with polio and cytomegalovirus have distinct DNA expression patterns, or "bar codes," that change over time. Although we have not processed a wide selection of infectious agents, we have evaluated approximately 60 distinct human tumor cell lines using a common control cell line. When we used these data for phylogenetic reconstruction, we found very good clustering with respect to the tissue of origin. Specific signatures are related to central nervous system tumors, kidney tumors, melanomas, and leukemias.

We would like to focus on the use of peripheral blood cells as a sort of infectious disease sensor. There are a number of reasons to believe that this may work. We have data from human lymphocytes harvested from whole blood (where one sample is exposed to interleukin-2 and the other is not) and we can demonstrate many changes in gene expression. To make this approach useful, we will need a broad range of gene array data from persons with known causes of illness.

